# Compete to Play: Trade-Off with Social Contact in Long-Tailed Macaques (*Macaca fascicularis*)

**DOI:** 10.1371/journal.pone.0115965

**Published:** 2014-12-31

**Authors:** Sébastien Ballesta, Gilles Reymond, Mathieu Pozzobon, Jean-René Duhamel

**Affiliations:** 1 Centre de Neuroscience Cognitive, Centre National de la Recherche Scientifique, Bron, France; 2 Département de Biologie Humaine, Université Lyon 1, Lyon, France; University of Bologna, Italy

## Abstract

Many animal species engage in various forms of solitary object play, but this activity seems to be of particular importance in primates. If playing objects constitute a valuable resource, and access to such objects is limited, a competitive context may arise. We inserted a unique toy within a mini-colony of long-tailed macaque (*Macaca fascicularis*) and compared their behaviors to sessions without playing object. An automatic color-based 3D video device was used to track the positions of each animal and the toy, and this data was categorized into 5 exclusive behaviors (resting, locomotion, foraging, social contact and object play). As expected, the delay to first access to the object reflected the hierarchy of the colony, indicating that a competition took place to own this unique resource of entertainment. In addition, we found that the amount of object play was not correlated with social or foraging behavior, suggesting independent motivational mechanisms. Conversely, object playing time was negatively correlated with idling time, thus indicating its relation to pastime activities. Interestingly, the amount of social contacts in the group was significantly reduced by the heightened competitive context, suggesting that competitors are more likely to be perceived as potential threat requiring caution, as shown in humans. Experimental manipulation of competitive contexts in primates reveals common mental processes involved in social judgment, and shows that access to valuable resources can be a sufficient cause for variations in group cohesion.

## Introduction

Competition over biological resources is crucial in shaping animal social structure, and is considered to be a key-mechanism of natural selection [1]. When commonly desired resources are not sufficient to fill the needs of all animals, each individual adopts social strategies to reach vital resources such as food, water or mate thus increasing their fitness [2]–[4]. Social animals such as primates were shown to respond to a competitive context by expressing their social status, and optimize their behavior to access to these limited resources while managing social interplays [5]–[10]. In humans, competition also occurs over non-vital resources, for instance during play. Object play behavior is also quite frequent in primates, both in wild and in captivity, and has been described as well in other species even in some invertebrates [11]–[16]. For primates in particular, playing with non-edible objects may even be considered as a genuine need, albeit non-physiological. Indeed, object-deprived laboratory environments significantly increase the risks of self-injury and stereotypies, which are undoubtedly linked to stress and anxiety [17]–[20], indicating that object play is necessary to animal welfare. In this study, we assess the existence of a competition over single playing resource in a group of long-tailed macaques (*Macaca fascicularis*), and measure its consequences on their social behavior.

By using a rugged object as the playable resource, instead of a consumable food item, we aimed to experimentally create a stable competition context within the mini-colony. Observations were compared to control situations without the presence of any playable object. The positions of the animals and of the object were simultaneously and individually tracked inside their home cage, using an novel automatic color-based 3D video tracking device [21], and positional data were used to categorize several mutually exclusive behaviors: resting, locomotion, foraging, social contact and object play.

## Material and Methods

### Animals

Four non-kin but group-housed male long-tailed macaques (*Macaca fascicularis*) (aged 3+/−0.15 years, weight 5.7+/−0.8) were housed in the animal facility of the Cognitive Neuroscience Center as a mini-colony inside a large enclosure (15 m^3^) favoring direct physical interactions, but also allowing to isolate the monkeys when needed through a system of sliding partitions. Animals were fed with monkey chow and received fresh fruits and vegetables. The cages were enriched with ground substrates to promote foraging, ropes, mirrors, toys, etc. The social hierarchy between monkeys was assessed thanks to the water bottle access test [22] (n = 5 sessions, Wilcoxon rank sum test, p<0.05) This study was approved by our local animal experimentation ethics committee (CELYNE) and used experimental procedures complying with the recommendations of the local authorities on Animal Care (Direction Départementale des Services Vétérinaires, Lyon, France) and the European Community standards for the care and use of laboratory animals [European Community Council Directive (1986), Ministère de l′Agriculture et de la Forêt, Commission Nationale de l′Expérimentation Animale]. This study was supervised by the Cognitive Neuroscience Center's Animal Welfare Committee.

### Automatic behavioral assessment

A custom-designed multi-camera 3D tracking system [21] was used to record and monitor the behavior of primates in their living space. This system can track the location of multiple animals in real-time, provided they wear a unique color marker (restraining collar or head-post cap). Animal positions (X, Y, Z) were estimated by triangulation from the set of image coordinates of their respective color targets when viewed by at least 2 cameras. Measurements for 4 animals and 1 colored toy were taken simultaneously at 15 Hz rate, with a nominal spatial accuracy of 1 cm. Individual behaviors were analyzed and classified using custom scripts written in Matlab R2010.

### Recording sessions

Data were acquired during 3-hour recording sessions started at 5 pm and finished at 8 pm before the gradual extinction of artificial lighting. Prior to each recording session, we made sure that no other objects were present inside the animal home cage, and we also removed the object from the animal's home cage the morning following the recording session. Sessions were alternated randomly between a condition where a single object was inserted just at the beginning of the recording (n = 28), and a condition where no object was provided (n = 17), Sessions with objects were carried out with the presence of either 3 (n = 18) or 4 animals (n = 10). Control sessions without objects were carried out with 3 animals (M2, M3, and M4) and compared to sessions with objects and 3 animals in order to assess the behavioral consequences of object introduction. Colored objects used in this study were commercial toys for pets (cats, dogs, and ferrets) or semi-professional circus gear, and never contained any kind of food. Although different toy objects were used in the study, the animals were able to observe the place where the toys were stored, thus likely leveling their intrinsic degree of novelty.

## Results and Discussion

**Figure 1:**
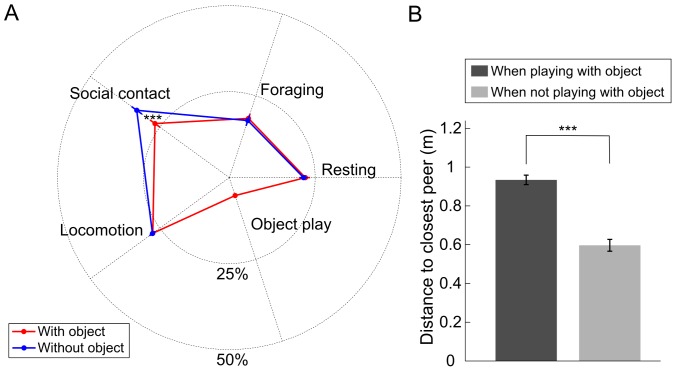
Impact of novel object introduction on monkeys' social and non-social activities (A) Radar plot of 5 mutually-exclusive individual activities of the mini-colony during recording sessions with (n = 18) or without (n = 17) prior introduction of a single toy (average and s.e.m.). (B) Distance to closest peer when a monkey was playing with the object or performing another activity (except social contact). All recording sessions with objects were used (n = 28). *** indicate significant differences (Wilcoxon rank sum test p<0.001).

**Figure 2:**
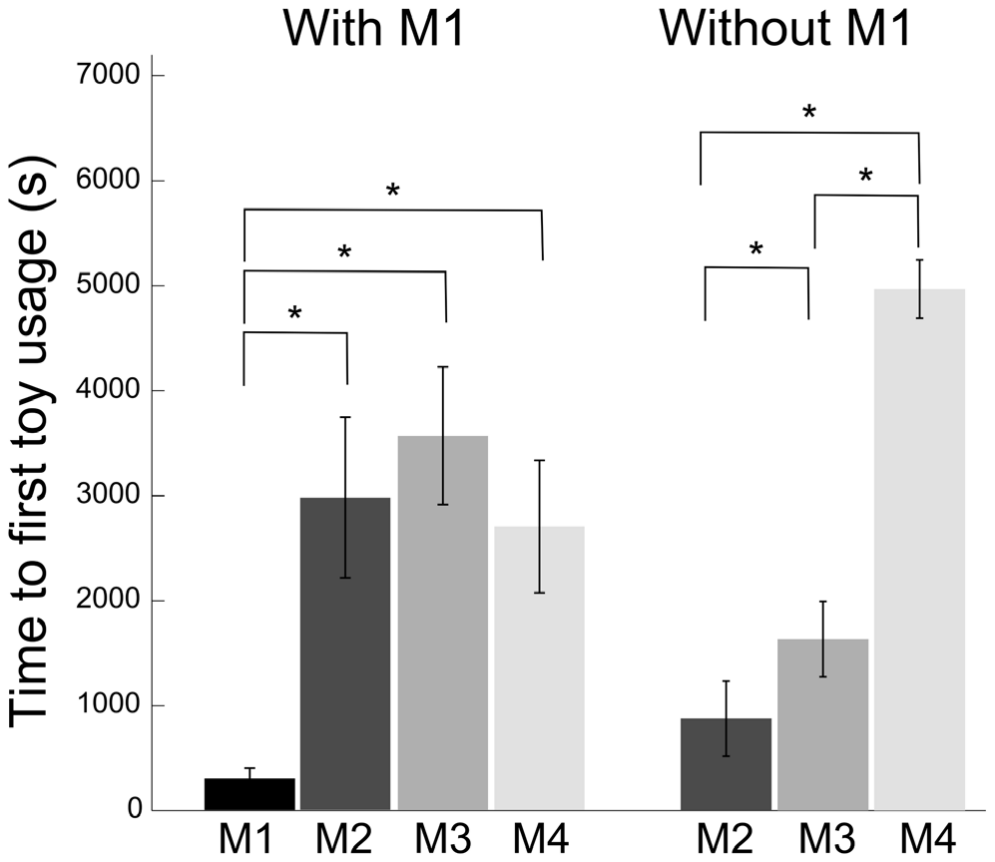
Hierarchy estimated from access to toys. Mean delay between toy introduction in the home cage and first toy interaction event lasting>10 s with M1 presence (left panel, n = 11) and absence (right panel, n = 18) in the group. * indicate significant pairwise differences (Wilcoxon rank sum test p<0.05). M1 designate the most dominant monkey and M4 the most subordinate monkey as established through the classical water access test (see Materials and Methods).

**Figure 3:**
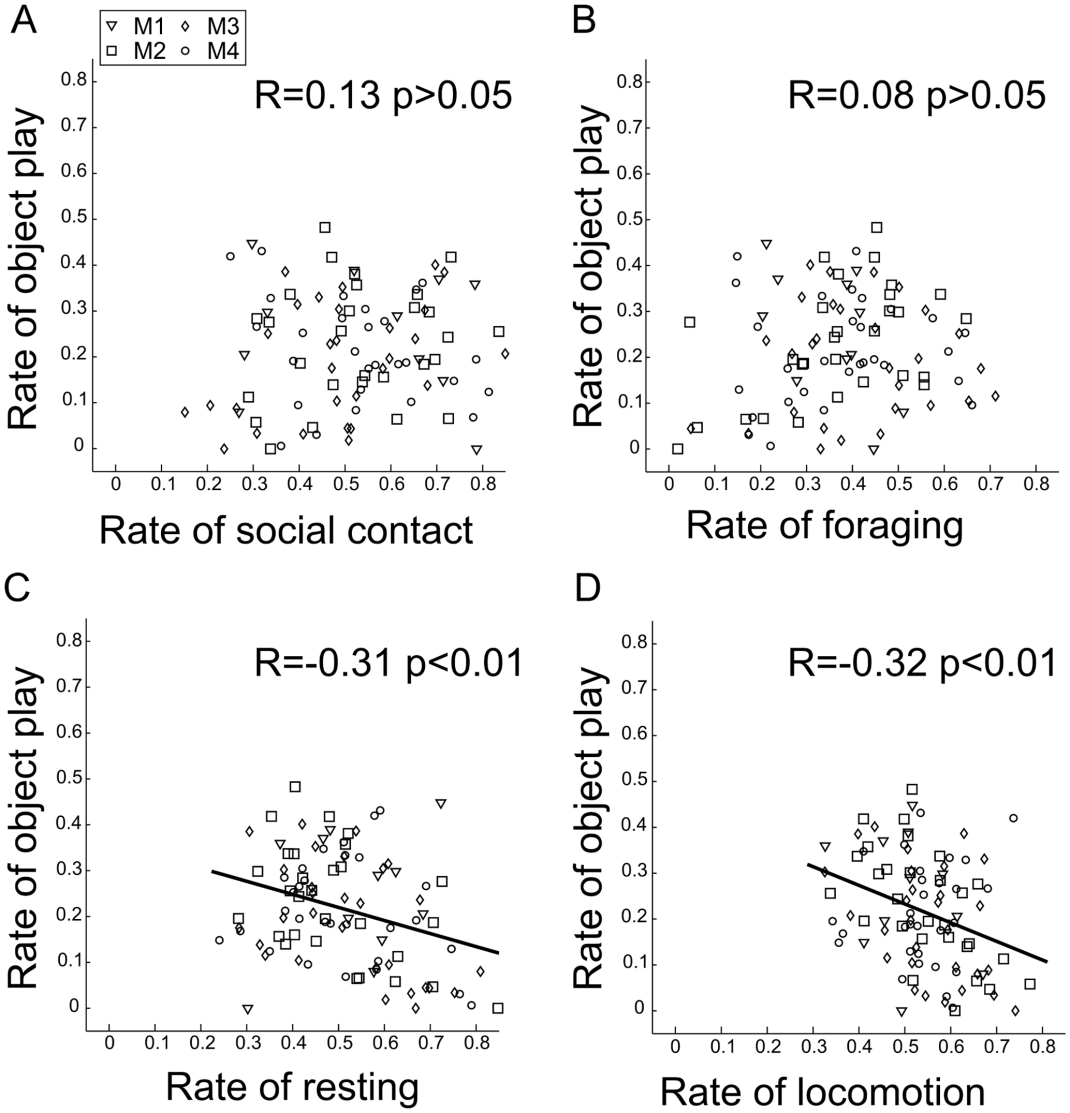
Intra-individual correlations between rate of object play and: (A) rate of social contact R = 0.13 p>0.05, (B) rate of foraging R = 0.08 p>0.05, (C) rate of resting R = −0.31 p<0.01, (D) rate of locomotion R = −0.32 p<0.01. All scores arcsine transformed. Symbols correspond to mean rate of a given behavior on a given day for a given monkey (M1-4: ▿, □, ◊, ○ respectively).

The behavior of the colony after a single object introduction was compared to control condition where no object was inserted within the home cage (Fig. 1A). On average, animals spent 5.5% (s.e.m. 0.7) in object play, a result comparable to other observations in wild environments [23]. By measuring the time between the introduction of the object and its first significant manipulation event (i.e. lasting over 10 s), we found that the dominant animals accessed the object sooner than subordinate ones (Fig. 2, Wilcoxon rank sum test, p<0.05), thus confirming the competitive context created by a single playing resource. By dividing the sessions in bins of 10 minutes, we found no effect of time on the occurrences of object play (Kruskal-Wallis test, p>0.05). Interestingly, the introduction of a single object significantly decreased the collective time budget spent in social contact (Fig. 1A, Wilcoxon rank sum test, p<0.05) from 33.3% (s.e.m. 1.9) to 26.7% (s.e.m. ±1.8), while not affecting any other measured behavior. During recording sessions with objects, the mean distance to the closest animal outside of social contact periods was significantly higher when an animal was handling the object than when it was not involved in object play behavior (Fig. 1B, Wilcoxon rank sum test p<0.001), indicating that the animals avoided their peers while playing with the object. Finally, for each monkey, the amount of object play was not significantly correlated with the amount of foraging or social behavior (Fig. 3A and B, p>0.05). This absence of relation suggests the existence of distinct motivational mechanisms for object play behavior. Conversely, significant negative correlations were found with resting and general locomotor behavior (Fig. 3C and D, linear regressions: R = −0.31, p<0.01 and R = −0.32, p<0.01).

### Competition interferes with social motivation

In our study, we have found that the simple introduction of a unique object inside an object-deprived environment significantly reduces the social behavior of our animals. We interpret this result as an effect of the competitive context created by the presence of a unique object, as the observed hierarchy in the group was found to be reflected closely by the order of access to the object. The increase in animal inter-distances observed when one was manipulating the object is consistent with studies showing that macaques have a sense of ownership and modulate their behaviors in accordance with the identity of the object owner [24], [25]. This effect might reflect an active avoidance by either the object owner or the observers, probably in order to prevent unnecessary conflicts triggered by the potential misinterpretation of a social approach. For the observer, approaching the player could be considered as a tentative of object appropriation, and for the player, keeping distance with others is the best strategy to quietly satisfy its playing needs. In any case, this result suggests that, when one is playing with an object, peers are more likely to be perceived as a threat. It could be argued that playing with an object somehow fulfills or compensates social needs, thus explaining the measured decrease of the amount of social contact. However, this interpretation would imply a proportional relation between social and object play behaviors, in contradiction with the observed absence of significant intra-individual correlation between the amount of object play and social contacts. This absence of such correlation may appear contradictory considering the fact that object introduction impedes social contact. However, the mean collective time budget allocated to the different scored activity (Fig. 1) and the correlation analyses (Fig. 3) reflect two distinct processes. The decreased social contact between group members in the presence of a novel object reveals the context of competition leading the monkeys to maintain a greater social distance. Hence, we would argue that a particular psychological state, incompatible with social interactions, is triggered by the competitive context created by the presence of a unique toy, and not by the individual playing activity itself. Meanwhile, the correlation results highlight the shared motivational mechanisms between object play and other behaviors, through the dependencies between their respective time budgets. The fact that the more a monkey plays, the less it engages in resting or locomotor activity, while playing is unrelated to searching for food or seeking social contact, suggests that the motivation beneath object play is independent from that underlying the latter two behaviors but that it is rather related to a kind of “idle motivation”, i.e. something one does when other needs are satisfied, and that such idling activity are not impacted by the competitive context.

In Humans, peers are perceived as potential threat when considered as competitors, thus eliciting caution, careful processing and conservative social judgments [26]. The measured decrease in social contact for macaques under competitive context could be thus interpreted as a manifestation of similar mental processes. In primates, competition goes beyond fighting for vital resources, and competing for other resources fulfilling psychological needs such as entertainment can as well induce a decrease in affiliative interactions. The biological and cognitive bases of competitive (and cooperative) interactions have been investigated extensively in Humans [26]–[28], however their evolutionary roots remain to be explored. For instance, comparative studies in distinct apes species have already shown differences in hormonal release in anticipation to a competitive interaction [29], which may be related to their respective social structure. Assessing such differences among the macaque genus would also be of particular interest to the phylogeny of primate social behaviors.

### Motivational nature of object play

Solitary object play is an activity that has been shown to be more prevalent in animals which diet relies on limbs and mouth use [30], and is probably correlated with the expertise required to extract nutrients from their food. For instance, fruit-eater primates seems to be more skilled and interested in object manipulation than non-fruit eaters [31], which may suggest a link between the motivation to play with objects and feeding behaviors. However, in our study, no correlation was found between the rate of object play and the rate of foraging. Conversely, significant correlations were found with idling time, suggesting that solitary object play may be better considered as a pastime activity [30], [32] having a motivational nature independent from food-seeking or social activities. This view is in accordance with the fact that object play is preferentially performed after having fulfilled physiological needs and in a secure environment [33]–[35]. As shown in the binning analysis, object play was uniformly performed across time, thus suggesting that the competitive context lasted throughout the whole recording session.

Solitary object play has an intrinsic value sufficient to justify competition and thus appears as an enjoyable activity, for several reasons, including the opportunity to destroy these objects ultimately. Many questions are still open about the ultimate and proximal bases of object play, as it adaptive value or it relation to tool usage remain under debate [36]–[41].

In conclusion, experimental manipulation of the competitive context using non-edible playable resources in non-human primates reveals shared mental processes involved in social judgment. Access to valuable resources can therefore be a sufficient cause for variations in group cohesion.

## Supporting Information

S1 Checklist **ARRIVE Checklist.**
(PDF)
